# Development of a Novel Multiplex PCR Assay to Detect Functional Subtypes of KIR3DL1 Alleles

**DOI:** 10.1371/journal.pone.0099543

**Published:** 2014-06-11

**Authors:** Jeanette E. Boudreau, Jean-Benoît Le Luduec, Katharine C. Hsu

**Affiliations:** 1 Immunology Program, Sloan-Kettering Institute for Cancer Research, Memorial Sloan Kettering Cancer Center, New York, New York, United States of America; 2 Department of Medicine, Memorial Sloan Kettering Cancer Center, New York, New York, United States of America; 3 Weill Medical College, Cornell University, New York, New York, United States of America; Karolinska Institutet, Sweden

## Abstract

Among NK cell receptor-ligand partnerships, KIR3DL1 and HLA-Bw4 demonstrate the greatest diversity; permutations of their allelic combinations titrate NK reactivity. Balancing selection has maintained distinct subtypes of KIR3DL1 alleles in global populations, implying that each may provide unique fitness advantages and variably influence disease processes. Though approaches exist for determining HLA-B allotypes, practical methods for identifying KIR3DL1 alleles are lacking. We have developed a PCR-based approach that identifies functional subtypes of KIR3DL1 alleles; it is suitable for research and may have clinical application. Six allele subsets were identified based on expression characteristics of the eleven most common KIR3DL1 alleles represented in reported populations. The remaining 62 low-frequency alleles were distributed into these groups based on sequence homology to coding regions. Subtype-specific SNPs were found in exons 3, 4, and 7, and used as priming sites for five multiplex PCR reactions. Genomic DNA derived from 175 unrelated donors and 52 related individuals from 6 families demonstrated >99.5% concordance between sequence-based typing and our novel approach. Finally, PCR-based typing accurately predicted NK phenotypes obtained by flow cytometry after staining with DX9 and Z27 monoclonal antibodies. This novel approach facilitates high-throughput analysis of KIR3DL1 allotypes to enable a broader understanding of KIR3DL1 and HLA-Bw4 interaction in health and disease.

## Introduction

Natural killer (NK) cells integrate activating and inhibitory signals to survey the health and identity of neighboring cells. To facilitate sensitivity to damaged cells without provoking autoagression, the activation potential of an NK cell is counterbalanced by its capacity for inhibition by “self” HLA class I ligands [1]. Among an array of receptors recognizing HLA are the killer immunoglobulin-like receptors (KIR), a polygenic, polymorphic family whose loci segregate separately from those of their ligands [2], [3]. Consequently, NK cells are “educated” according to their ability to recognize “self” HLA: those expressing KIR that can be inhibited by local HLA are “licensed” and activate rapidly upon contact with cells whose expression of HLA is low or absent [4]–[6]. “Unlicensed” NK cells are hyporesponsive to “missing self”, but may be activated under inflammatory conditions [4], [5].

Downregulation of HLA, common in viral infections, prompts “missing self” reactivity in NK cells. Indeed, the co-expression of inhibitory KIR3DL1 and cognate HLA-Bw4 has been associated with superior control of HIV infection, where HLA-B expression is known to be down-regulated by the viral *nef* protein [7], [8]. Though beneficial in some circumstances, the sensitivity to inhibition that engenders licensing may be a liability in pathologies where HLA is maintained or upregulated. The presence of unlicensed NK cells is associated with decreased leukemic relapse following hematopoietic stem cell transplantation [6], [9], protection against Crohn’s disease [10], and beneficial outcomes in patients with neuroblastoma receiving anti-GD2 therapy [11]. Thus, NK licensing cannot be uniformly classified as beneficial or harmful in disease processes, rather, education diversifies NK responsiveness on the population level to manage a variety of evolutionary pressures.

Among receptor-ligand pairs, the most diverse is that of HLA-Bw4 and KIR3DL1 [12]. The KIR3DL1 locus encodes both inhibitory and activating (KIR3DS1) alleles that can be subdivided based on the extent to which they are expressed on the cell surface and bound by DX9 and Z27 monoclonal antibodies [13], [14]. Inhibitory members of the KIR3DL1 family are bound by both antibodies and expressed on the cell surface at high and low densities, or are retained in the endoplasmic reticulum and undetectable by surface staining [13], [15]. Conversely, the activating KIR3DS1 subtype is weakly bound by Z27 and cannot be detected by DX9 [16], [17]. The frequency of KIR3DL1^+^ NK cells within the NK repertoire additionally varies by allele, where low surface density also predicts for low KIR3DL1^+^ NK frequency, and high-density alleles are expressed at higher and more variable frequencies [14], [18].

Balancing selection has maintained diversity among KIR3DL1 low expressing alleles (KIR3DL1-low), KIR3DL1 high-expressing alleles (KIR3DL1-high) and KIR3DS1 in at least 28 distinct populations worldwide, strongly selecting for six residues implicated in contact with HLA-Bw4 molecules [19]. HLA-Bw4 molecules are dichotomized based on the amino acid present at position 80: isoleucine or threonine. Isoleucine-encoding (80I) Bw4 molecules more readily inhibit KIR3DL1-high compared with KIR3DL1-low alleles, and those encoding threonine at position 80 (80T) confer relatively weaker inhibition via both KIR3DL1-high and -low receptors [14], [20], [21]. Consequently, NK cells’ sensitivity to inhibition, and by extension, NK licensing may vary even among receptor-ligand partnerships based on the specific alleles present.

Sequence-based typing of KIR3DL1 in patients with HIV provided the first evidence that compound KIR3DL1 and HLA-B allotypes variably influence disease progression. In patients expressing 80I isoforms of HLA-Bw4, KIR3DL1-high is associated with delayed AIDS onset compared with other isoforms of HLA-B. Additional partnerships of KIR3DL1 and HLA-Bw4 provide intermediate protection, and patients lacking HLA-Bw4 epitopes demonstrated the most rapid progression to AIDS [8]. Collectively, these findings imply that a range of anti-HIV NK reactivity exists and may reflect variable NK education conveyed by KIR3DL1 and HLA-B allelic diversity. Assessment of KIR3DL1 and HLA-B compound allotypes may therefore assist in determining prognosis for patients with HIV and, by extension, other diseases where NK reactivity correlates with disease outcomes.

Donor HLA-B allotypes are readily determined by sequence-specific and histologic approaches; however, similar high-throughput methods for classifying KIR3DL1 alleles have been lacking. We describe a novel approach that divides KIR3DL1 alleles into functional subtypes based on the characteristics of their surface expression and sensitivity to inhibition by HLA-B. This multiplex PCR assay delivers medium-resolution typing and yields results that are consistent with genomic sequencing and segregation among multi-generational family members. This approach provides an efficient, cost-effective alternative to sequence-based typing and bead arrays and can be performed using standard laboratory materials and equipment. We anticipate that KIR3DL1 classification will facilitate a broader understanding of these alleles as they relate to NK cell function and influence disease processes.

## Materials and Methods

### Sequence Alignments and Primer Design

All KIR3DL1 allele-coding sequences from the EMBL-EBI IPD KIR database (http://www.ebi.ac.uk/ipd/kir/alleles.html) were included in our alignment analyses, and suballeles differing exclusively within intronic regions were classed with their canonical allele. Gene alignments and phylogenetic analyses were performed using MacVector software version 12.0.

### Grouping Strategy and Primer Design

**Figure 1:**
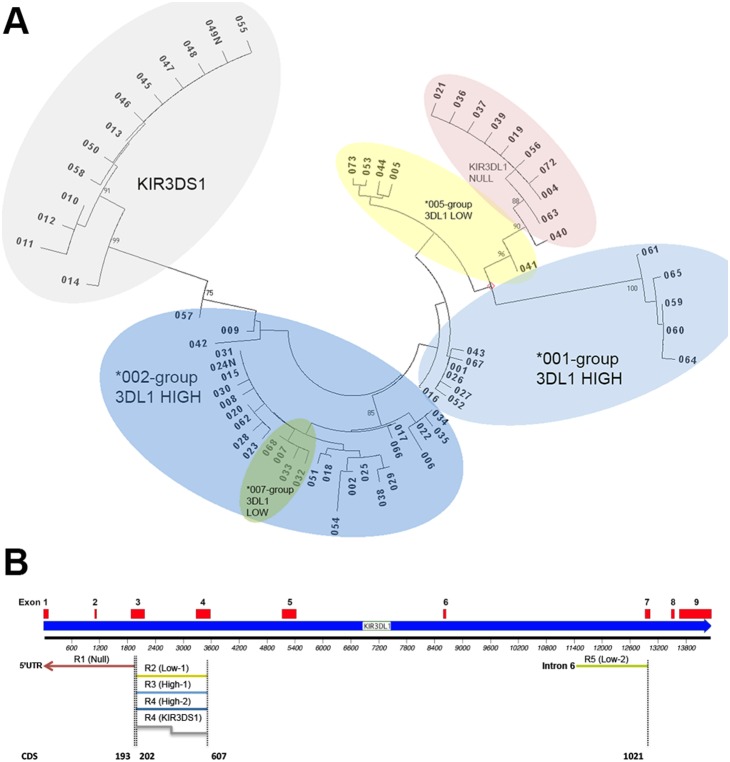
Division of alleles by coding sequence homology. (A) All available coding sequences for KIR3DL1 alleles were used to create a neighbor-joining phylogenetic tree. Confidence intervals were estimated using 1000 bootstrap replicates. (B) Schematic representation demonstrating the location of SNPs targeted in each PCR reaction. CDS numbers correspond with exon coding sequences, and their location within the KIR3DL1 gene is shown.

KIR3DL1 frequencies determined by genomic sequencing in a cohort of 426 healthy donors of primarily European ancestry [22] revealed that 11 alleles were present with >1% frequency each and collectively comprised 97.5% of all 852 KIR3DL1 alleles in the donors (Table 1). The remaining low-frequency alleles were assigned to groups based on sequence homology with high-frequency alleles in the exon coding regions following unsupervised phylogenetic analysis (MacVector) (Figure 1 and Table 2). The 11 alleles were then grouped into 6 functionally important subtypes, defined by expression level and Bw4 specificity. Primer pairs targeting SNPs present in each subtype group were identified and their specificity for KIR3DL1 was confirmed using NCBI primer blast. To provide an internal control for DNA quality, a 650 bp control band derived from a conserved region of HLA-DR was multiplexed into each reaction [23]. Specific primer sequences and PCR conditions are shown in Tables 3 and 4, respectively.

**Table 1:** KIR3DL1 allelic frequency in 426 donors determined by KIR3DL1 sequencing.

KIR3DL1 allele	Frequency (%)
KIR3DS1*013	19.80
High*001/*016	18.80
Null*004	15.80
Low*005	14.00
High*002	11.50
High*015	6.20
High*008	5.50
Low*007	2.70
Unknown*009	1.90
High*020	1.30
*019	0.94
*049	0.47
*052	0.35
*014	0.12
*018	0.12
*030	0.12
*053	0.12
*072	0.12
*073	0.12

**Table 2:** Distribution of KIR3DL1 alleles and primer target sites.

	KIR3DL1 alleles		Exon 3		Exon 4	Exon 7
KIR3DL1	High frequency	Low frequency	Position	Position	Position	Position
Subgroup	alleles	alleles	193	202	607	1021/1022
Null	KIR3DL1*004	KIR3DL1*019, *021,	G	A	T	T/G
		*036, *037, *039,				
		*040, *056, *063				
		*072				
Low	KIR3DL1*005	KIR3DL1*041, *044,	A	A	T	C/A or T/G
		*053				
High	KIR3DL1*001,	KIR3DL1*026, *027,	A	A	C	C/A or T/G
	*016	*043, *052, *059				
		*060, *061, *064				
		*065, *067, *075				
High	KIR3DL1*002,	KIR3DL1*006, *017,	A	G	C	C/A
	*015, *008,	*018, *022, *023,				
	*009, *020	*024N, *025, *028				
		*029, *030, *031				
		*034, *035, *038				
		*042, *051, *054,				
		*057, *062, *066				
		*074, *076, *077				
Low	KIR3DL1*007	KIR3DL1*032, *033,	A	G	C	C/G
		*068				
KIR3DS1	KIR3DS1*013	KIR3DS1*010, *011,	A	G	C	C/A
		*012, *014, *045				
		*046, *047, *048				
		*049N, *050, *055				
		*058				
Unknown		KIR3DL1*042	A	G	T	C/A
Unknown		KIR3DL1*073	A	A	A	C/A

**Table 3:** PCR primers and reaction conditions.

Target KIR3DL1 subtypes(most common allele)	Name	Sequence (5′->3′)	Amplicon size (bp)
Null (KIR3DL1*004)	ConsF^a^	ATCCTGTGCGCTGCTGAGCTGAG	2019
	193G-R	CATGGAAGATGGGAATGTGGATTCC	
Low-1 (KIR3DL1*005)	202A2-F	CAATTTCATGCTATACAAAGAAGACA	1573
	607T-R	GGGRGCTGACAACTGATAGGA	
High-1 (KIR3DL1*001)	202A3-F	GCTATACAAAGAAGACAGAATCCACA	1573
	607C-R	GGGAGCTGACAACTGATAGGG	
High-2/KIR3DS1	202G-F	CAAAGAAGACAGAATCCACG	KIR3DL1: 1573
(KIR3DL1*002/KIR3DS1*013)	607C-R	GGGAGCTGACAACTGATAGGG	KIR3DS1: 1933
Low-2 (KIR3DL1*007)	int6-F	CAGAGATCTGTGCCAGC	1408
	1021/22-S-R	GAGGTCCCAATCAGAACG	
HLA-DR (internal control)*	FDRA-360^a^	GAGGTAACTGTGCTCACGAACAGC	607
	RDRA-633^a^	CACGTTCTCTGTAGTCTCTGGG	

a internal control primers and ConsF have been described previously (2, 23).

**Table 4:** PCR reaction conditions.

	Target KIR3DL1 subgroup		uM	Annealing	Extension	Numberof cycles
Reaction	(most common allele)	Primers	primers	temp	Time (min)	
1	Null (KIR3DL1*004)	ConsF	0.795	66.9°C	3∶30	30
		193G-R	0.795			
		FDRA-360	0.040			
		RDRA-633	0.040			
2	Low-1 (KIR3DL1*005)	202A2-F	0.795	65.6°C	3∶30	30
		607T-R	0.795			
		FDRA-360	0.040			
		RDRA-633	0.040			
3	High-1 (KIR3DL1*001)	202A3-F	0.795	67.0°C	3∶45	35
		607C-R	0.795			
		FDRA-360	0.173			
		RDRA-633	0.173			
4	High-2/KIR3DS1	202G-F	0.795	64.2°C	4∶00	35
	(KIR3DL1*002/KIR3DS1*013)	607C-R	0.795			
		FDRA-360	0.040			
		RDRA-633	0.040			
5	Low-2 (KIR3DL1*007)	int6-F	0.795	64.2°C	3∶45	35
		1021/22-	0.795			
		C/G-R				
		FDRA-360	0.086			
		RDRA-633	0.086			

### Optimization of PCR Reactions

PCR reaction conditions were optimized and validated using Applied Biosystems PCR System 9700 and Eppendorf Mastercycler proS thermocyclers, and further validated using the ProFlex PCR system (Life Technologies). 50 ng of DNA was included in each 25 µL reaction, prepared with Taq polymerase, dNTP and PCR buffer according to the manufacturer’s recommendations (Roche, Nutley, NJ). Control and allele-specific primer concentrations were optimized independently for each reaction to provide maximum specificity and consistent allele group-specific amplification (Tables 3 and 4). PCR reactions were optimized using gradients of annealing temperature that ranged from 52–72°C, and the highest annealing temperatures that allowed specific and sensitive SNP detection were selected for subsequent optimization. All PCR reactions were further validated on three different models of PCR machines to ensure generalizability.

PCR products were analyzed by electrophoresis on 1.5% agarose gels for 40 min at 125 V. Control bands (650 bp) confirmed DNA quality. Specific product sizes ranged from 1.5–2.0 kB (Table 3).

### Cells, DNA Sources and Preparation

DNA samples were extracted using Blood mini kits according to the manufacturer’s instructions (Qiagen, Valencia, CA). EBV-immortalized BLCL lines derived from multi-generational families were produced by the Centre d’Etude Polymorphisme Humaine (CEPH), and kindly provided by Dr. Bo Dupont (MSKCC). Cell lines were maintained in RPMI-1640 media containing 100 U/mL penicillin/streptomycin and 10% FBS. DNA from unrelated human volunteer hematopoietic stem cell donors was provided by the National Marrow Donor Program (NMDP) Research Repository and were collected under an NMDP Institutional Review Board informed research consent (IRB #1991-0002) [24]. KIR3DL1 alleles were identified by sequence-based typing, as previously described [22]. PBMCs were collected from healthy human donors at MKSCC following approval from the MSKCC Institutional Review Board (IRB #95-054) and donors provided informed, written consent. Additional PBMCs were isolated from buffy coats obtained from healthy volunteer donors via the New York Blood Center (http://nybloodcenter.org/). The MSKCC IRB waived the need for additional research consent for anonymous NYBC samples.

### Phenotypic Analysis by Flow Cytometry

KIR3DL1/S1 expression was detected on NK cells using the following monoclonal antibodies: anti-KIR3DL1 (clone:DX9, Brilliant Violet 421, Biolegend, San Diego, CA, USA), anti-KIR3DL1/S1 (clone:Z27, APC, Beckman Coulter, Brea, CA, USA), anti-CD56 (clone:N901, ECD, Beckman Coulter) and anti-CD3 (OKT3, Brilliant Violet 650, Biolegend). Dead cells were excluded by staining with DAPI and NK cells were classified as CD3-CD56+. All FACS analyses were performed on an LSR Fortessa (Beckton Dickenson, San Jose, CA, USA) and analyzed using FlowJo 9.7 software (Treestar, Ashland, OR, USA).

## Results

### Identification of KIR3DL1 Allele Subtypes

KIR3DL1 alleles were previously determined by sequence-based typing in a cohort of 426 healthy individuals acting as donors for hematopoietic stem cell transplantation [22]. The results and distribution of KIR3DL1 alleles in this group are shown in Table 2. Eleven alleles accounted for 97.5% of all identified alleles in this primarily Caucasian cohort and in worldwide populations (allelefrequencies.net). The 11 alleles were then grouped into six functionally defined subtypes, based on known expression levels and Bw4 specificity [12], [18]: null (*004), 005-group low (*005), 007-group low (*007), 001-group high (*001, *016), 002-group high (*002, *008, *009, *015, *020) and activating KIR3DS1 (*013). To assign the alleles represented at frequencies below 1% to functional subtypes, we performed unsupervised hierarchical clustering based on their coding sequences (Figure 1A). By this approach, low-frequency alleles were clustered according to their evolutionary relationships to high-frequency alleles [12]. We therefore focused the design of our allele typing strategy on the six functional groups defined above, recognizing that low-frequency alleles were unlikely to form additional unique subsets (Table 2).

The coding sequences of all KIR3DL1 alleles were aligned, stratified by their phylogenetic groupings and queried for single nucleotide polymorphisms (SNPs) which identify allelic subsets. Two polymorphic regions, present in exons 3 and 4, collectively allowed the division of KIR3DL1 alleles into five mutually exclusive groups distinguishable by four PCR reactions (Tables 2 and 3, Figure 1B).

The null group of alleles is identified using a reverse primer that targets a subtype-defining A→G SNP at position 193, coupled with a forward primer that targets a conserved region in the 5′ UTR [2]. The remaining subtypes did not have a single group-defining SNP, reflecting their evolutionary origins by recombination [25]. The KIR3DL1*001 subtype alleles combine the D0 domain (exon 3) found in the *005-group low alleles with D1 domain (exon 4) found in the *002-group of high alleles. To distinguish these three subtype groups, we selected polymorphic sites in each of exons 3 (position 202) and 4 (position 607), and employed two site-specific primers in each reaction. Neither the forward nor reverse primer was individually subtype-specific, but the combination of both was subtype-specific.

The SNPs used to target the KIR3DL1*002 group are common among three subtypes of alleles: the KIR3DL1*002-group high, KIR3DL1*007-group low and the activating KIR3DS1 alleles, and generate two distinct amplicons in the PCR reaction. Compared with the inhibitory alleles, intron 3 is 360 bp longer in the KIR3DS1 allele group; hence, activating and inhibitory receptors can be distinguished based on PCR product size. KIR3DL1*007, and its highly homologous low-frequency alleles, *032, *033, and *068, are identical to KIR3DL1*015, a high-expressing allele amplified among the *002-group, in their extracellular domains and intron sizes, but vary at positions 1020 and 1021 within the transmembrane-coding region. Given that KIR3DL1*007 is expressed with low surface density and conveys distinctly weaker inhibitory signals than KIR3DL1*002 [14], we designed a fifth PCR reaction to target positions 1020–1021 and to distinguish KIR3DL1*007-low from the KIR3DL1-*002-high (Tables 2 and 3, Figure 1B). A forward primer was designed to target a region conserved in the upstream intron. The expected reactivity patterns for each potential combination of KIR3DL1 subgroups are illustrated in Table S1.

### Optimization and Validation of PCR-based Medium-resolution KIR3DL1 Allotyping

**Figure 2:**
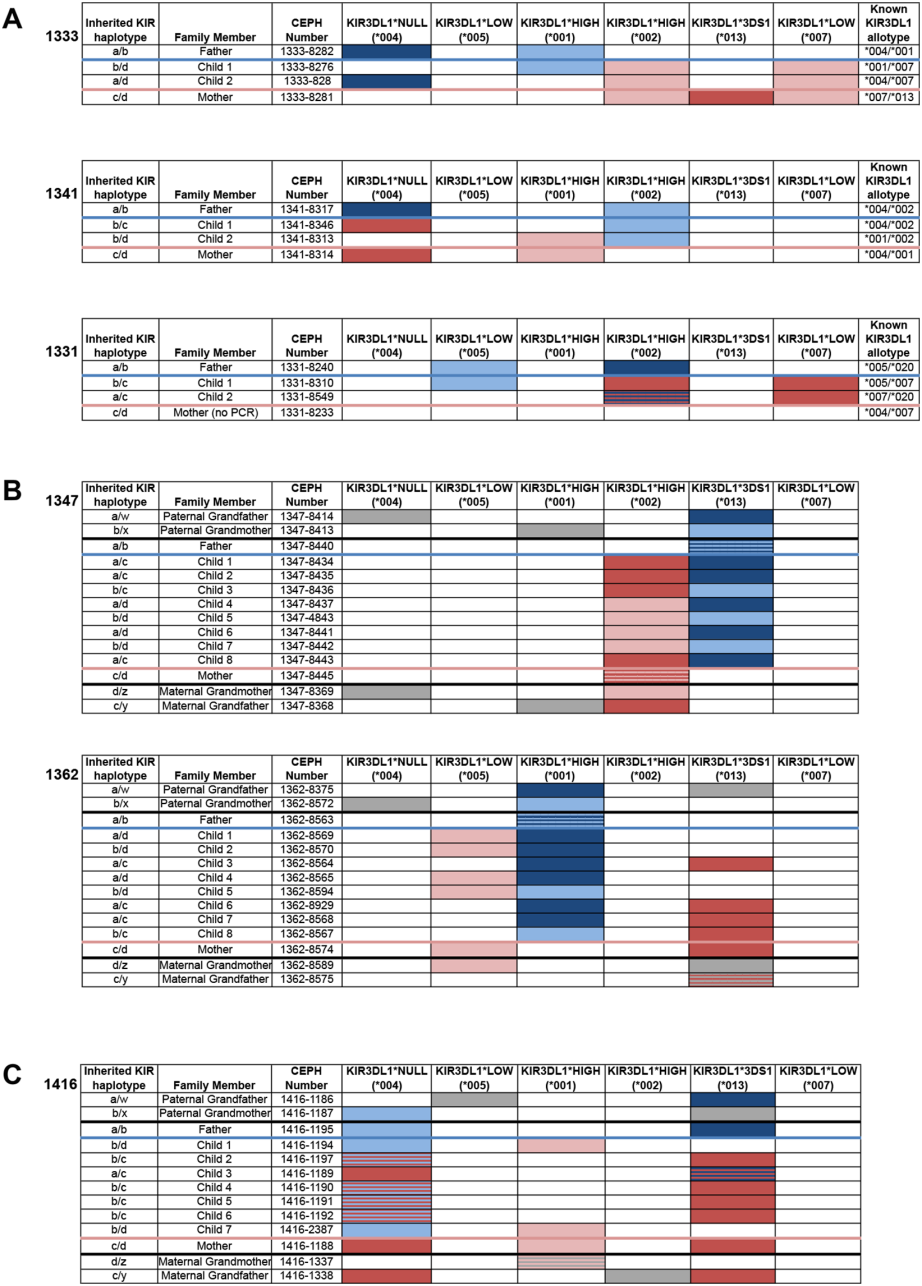
Multiplex PCR assessment accurately predicts KIR3DL1 allotypes. (A) KIR3DL1 subtyping was completed for three CEPH parent-child quartets and matched previous KIR3DL1 allele typing [26], [27]. Paternal alleles are shown in blue; maternal in red. (B) KIR3DL1 subtype analysis of three generations of CEPH family individuals demonstrates Mendelian inheritance. Non-inherited alleles present in grandparents are indicated by grey shading. (C) Analysis of CEPH family 1416 demonstrates inheritance of a polyallelic haplotype, encoding both KIR3DS1 and a KIR3DL1-null allele.

Immortalized B cell lines from the Centre d’Etude Polymorphisme Humaine (CEPH) were first employed to optimize our reaction conditions. We selected four family members, two parents and two children, from each of three families. These quartets’ KIR3DL1 alleles had been previously determined by sequence-based typing [26], [27]; our KIR3DL1 subtyping yielded results that had 100% concordance these characterizations (Figure 2A).

To confirm and extend these findings, we isolated and genotyped DNA from three additional extended CEPH families, each containing four previously-genotyped members plus grandparents, and additional children whose KIR3DL1 alleles had not been previously determined (Figure 2B). KIR3DL1 subtypes were assayed using our typing method, and the typing results were then compared against the known inheritance patterns of the KIR3DL1 loci [reference [2] and unpublished findings). As expected, KIR3DL1 alleles demonstrated the same inheritance patterns as their KIR haplotypes, further confirming the accuracy of our KIR3DL1 allele subtype approach.

Unequal crossing over has been described to uncommonly lead to an increase or decrease in the number of KIR3DL1 alleles present in a single haplotype [28], [29]. In CEPH family 1416, a KIR haplotype was found to contain two KIR3DL1 alleles, leading to some family members having 3 KIR3DL1 alleles in total [27], [28]. The extra allele was not clearly evident by allele typing defined using sequence-specific oligonucleotide priming [26]. By our multiplex PCR approach, the extended haplotype was identified in child 1416-1197 and could furthermore be traced to his mother and maternal grandfather and found in three siblings (Figure 2C).

### Validation of KIR3DL1 Allotyping in Unrelated Individuals

**Figure 3:**
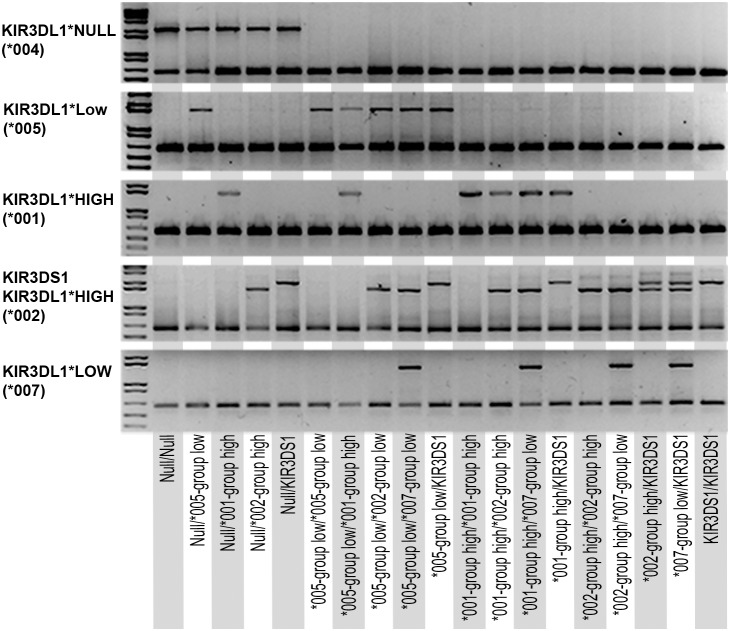
Amplification of specific KIR3DL1 functional subtypes. KIR3DL1 subtypes among unrelated donors were assessed using the multiplex PCR typing method. A panel of DNA representing homozygosity for each subtype or combination of two subtypes, is shown.

KIR3DL1 allotypes were previously determined by sequence-based typing in a cohort of 426 healthy, unrelated donors for hematopoietic stem cell transplantation [22]. To confirm the validity of our typing method, we assayed the KIR3DL1 subtypes in a subset of 181 samples. Our results demonstrated 99.5% concordance with sequence-based typing (Table S2). Using a panel of donors designed to amplify each allelic subtype group alone and in combination with each of the other subtype groups, we were able to demonstrate specific amplification of the intended and known allele subtypes for each reaction (Figure 3).

### KIR3DL1 Allele Subtypes Predict NK Phenotype

**Figure 4:**
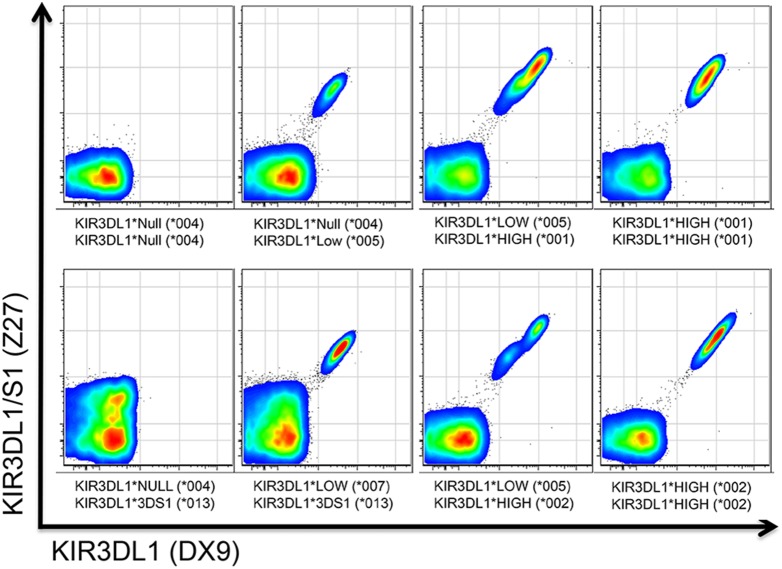
KIR3DL1 subgroup typing accurately predicts NK phenotypes by multi-parameter flow cytometry. NK cells from eight representative healthy donors were surface-stained with DX9 and Z27 antibodies. KIR3DL1 subtypes identified by the multiplex typing method have known expression characteristics and they are indicated below each of the eight flow cytometry plots shown. KIR3DL1 subtyping and NK phenotypes were consistent for all 104 individuals tested.

KIR3DL1 alleles were initially characterized based on differences in surface density, as determined by staining with the DX9 antibody. Surface staining using the Z27 antibody, which binds both KIR3DL1 and KIR3DS1, in combination with DX9 antibody allows categorization of KIR3DL1^+^ NK cells into null, low, high and KIR3DS1 surface expression groups [13], [14], [16], [17]. We harvested PBMC from healthy donors to ascertain whether our KIR3DL1 subtyping approach could similarly predict NK phenotypes. As shown in Figure 4, each allele detected by PCR in a given donor correctly predicted the presence of an NK population with the anticipated phenotype. Specifically, PCR-based typing for KIR3DL1-high and –low alleles predicted high and low density receptor expression, respectively, and bound both Z27 and DX9 antibodies as previously demonstrated [13], [17]. KIR3DS1 was present as a Z27+DX9- population; and as expected, the product of the null allele was not observed on the cell surface.

## Discussion

Among partnerships of KIR and HLA class I ligands, KIR3DL1 and HLA-Bw4 demonstrate extremely high polymorphism [12], and combinations of allelic subtypes have been found to influence the outcomes of infectious and chronic disease. HLA-B alleles are readily determined by commercial assays, but the classification of KIR3DL1 alleles currently requires lengthy, costly, and labor-intensive procedures including long-range sequencing or mRNA-based oligonucleotide primer arrays [14], [26], [30]. We have designed an intermediate resolution PCR-based typing method that identifies subtypes of KIR3DL1 alleles based on known functional characteristics including expression density and ligand specificity. Although the method was designed based on the phenotypes of the eleven overwhelmingly most common alleles for which functional differences have been described [14], [20], [22], [31], [32], this approach identifies 71 of the 73 alleles for which coding sequences are published, categorizing them in a manner consistent with their phylogenetic associations to their well-described homologs [12]. Using genomic DNA derived from healthy donors and immortalized cell lines from multi-generational families, we demonstrate that this novel method produces results that are consistent with sequence-based typing. Finally, we confirm that allele typing in this manner accurately predicts KIR3DL1^+^ NK cell phenotypes, as determined by staining with DX9 and Z27 monoclonal antibodies.

We identified six subtypes of alleles based on the phylogeny, phenotype and signaling characteristics of 11 high-frequency alleles collectively representing nearly 98% of all alleles identified in a cohort of 426 individuals. The validity of this approach was subsequently tested by PCR analysis in a subset of these donors. The majority of these individuals were of European ancestry, and we have not empirically validated this method in a more heterogeneous population. An online compendium of KIR3DL1 allelic distributions (available at allelefrequencies.net) nevertheless indicates that the 11 most frequent alleles in our sample cohort represent the vast majority of those found in worldwide populations. Moreover, the regions targeted by our assay are highly conserved among KIR3DL1 subtypes; thus, we expect that even low-frequency alleles will be accurately classified by our method. In agreement with this assertion, our sample cohort contained 11 low-frequency alleles that amplified as predicted by their gene sequences and homology to high-frequency alleles. It should be noted that within allele subgroups, high-frequency alleles cannot be distinguished by the sequences of the amplicons generated.

In addition to the activating KIR3DS1 subtype typified by KIR3DL1*013, phylogenetic subtypes of alleles represented by KIR3DL1*004 (Null), KIR3DL1*005 and KIR3DL1*007 (Low), and KIR3DL1*001 and KIR3DL1*002 (High) are identified by our typing method. It should be noted that KIR3DL1*007 is identified in two separate PCR reactions, one of which also identifies the KIR3DL1*002 allele group. Therefore, a positive result for both reactions could indicate heterozygosity for a KIR3DL1*002-group allele and KIR3DL1*007 or homozygosity for KIR3DL1*007, the latter calculated to occur in less than 0.01% of genotypes in our largely Caucasian cohort. Notably, however, frequencies of KIR3DL1*007 greater than 10% have been reported in subpopulations of African and Asian descent [19]. Thus, further clarification of individuals whose KIR3DL1 subtypes are exclusively within the KIR3DL1*002 and *007-groups may be desired in some studies. Sequencing of exon 7, where positions 1020–1021 are different in KIR3DL1*007 vs the KIR3DL1*002 group, will permit distinction between KIR3DL1*002/*007 heterozygosity and KIR3DL1*007 homozygosity. High- and low-expression subtypes, previously determined by DX9 and Z27 staining [13], [17], are each the products of two distinct subclades of KIR3DL1. Overlapping sequence homology and combinatorial diversity in the coding regions for D0 and D1 complicated our assay design, requiring four independent PCR reactions for identification of specific high- and low-expressing subtype groups. However, despite their convergence on a similar phenotype, subsets of high and low alleles may still differ by interaction with HLA-Bw4 ligand and/or representation among the NK cell repertoire.

Though expressed with a low density similar to KIR3DL1*005 [13], KIR3DL1*007 harbors an Ig-like domain identical to that of the high-expressing KIR3DL1*015 and similarly binds Bw4-80I tetramers preferentially over Bw4-80T [32], [33]. Variations among coding sequences of the high subtype groups KIR3DL1*001 and *002 may also impact their respective functional capacities despite their phenotypic commonality. KIR3DL1*001 combines the D0 domain of KIR3DL1*005 with the D1 and D2 domains of KIR3DL1*002, where residues from all three domains are known to influence binding to the Bw4 epitope [25]. Both KIR3DL1*001 and KIR3DL1*002 group alleles are highly responsive to inhibition by Bw4-80I alleles; however, specific alleles within these subtype groups still create distinct inhibitory patterns [14], [21]. Collectively, these findings imply that the typical description of KIR3DL1 alleles based on surface density may incompletely predict function. By permitting classification of more refined subtype groups that distinguish the subsets of high (*001 vs. *002) and low (*005 vs. *007) alleles, our method may facilitate a broader assessment of KIR3DL1-mediated contributions to NK function in health and disease.

The genetic loci for KIR and HLA are located on separate chromosomes and consequently segregate independently. NK cell education, as a consequence of interaction between KIR and their HLA ligands, will vary even among related individuals. The allelic diversity of KIR3DL1 and HLA-B has been maintained in global populations as this receptor-ligand pair has co-evolved, implying that they convey important and diverse fitness advantages [19]. Patients with HIV have provided the first evidence that KIR3DL1 and HLA-B compound allotypes can variably influence disease outcomes, whereby highly-inhibitory combinations of Bw4-80I and KIR3DL1-high alleles best protect against progression to AIDS [8]. Whether these allotypic combinations are similarly protective against other viral infections such as human papilloma virus (HPV) and Epstein-Barr virus (EBV) which, like HIV, induce downregulation of HLA-B, remains to be determined [7], [34]. Conversely, in pathologies where HLA expression persists or is upregulated, the presence of receptor-ligand subtypes predictive for poor inhibition may prove to be more beneficial.

KIR3DL1 allele subtyping by PCR is a simple, affordable, alternative to methods that currently rely on sequencing to identify KIR3DL1 subtypes. This method therefore facilitates assessment of KIR3DL1 alleles without requiring specialized equipment and personnel. Consequently, this technique will enable investigations of how KIR3DL1 polymorphisms impact NK cell function and various disease processes. Associations between KIR3DL1 and HLA-B alleles already have prognostic implications in patients with HIV infection [8]. Similar studies in other disease states may further extend the clinical relevance of KIR3DL1 and HLA-B interactions. We anticipate that assessment of KIR3DL1 subtypes will become increasingly relevant in clinical applications as their interplay with HLA-B alleles and consequent roles in immune function are described.

## Supporting Information

Table S1 **Expected reactivity patterns of KIR3DL1 subgroups.**
(DOCX)

Table S2 **Sensitivity and specificity of KIR3DL1 allele subtype assessment.** High frequency KIR3DL1 alleles are shown in bold.
(DOCX)
