# Supplementary material for: Development of a Novel Multiplex PCR Assay to Detect Functional Subtypes of KIR3DL1 Alleles
Source: PLoS One. 2014 Jun 11;9(6):e99543. doi: 10.1371/journal.pone.0099543 (PMC4053526; doi:10.1371/journal.pone.0099543)
Supplement: Table S1 — Expected reactivity patterns of KIR3DL1 subgroups. (DOCX) [file pone.0099543.s001.docx]

**Supplementary Table S1.** Expected reactivity patterns of KIR3DL1 subgroups.

|  | KIR3DL1 genotype | Null (KIR3DL1*004) | Low-1 (KIR3DL1*005) | High-1 (KIR3DL1*001) | High-2 (KIR3DL1*002) | KIR3DS1*013) | Low-2 (KIR3DL1*007) |  |
| --- | --- | --- | --- | --- | --- | --- | --- | --- |
|  | PCR Reaction | 1 | 2 | 3 | 4 | 4 | 5 |  |
|  | *004/*004 | X |  |  |  |  |  |  |
|  | *004/*005 | X | X |  |  |  |  |  |
|  | *004/*001 | X |  | X |  |  |  |  |
|  | *004/*002 | X |  |  | X |  |  |  |
|  | *004/*013 | X |  |  |  | X |  |  |
|  | *004/*007 | X |  |  | X |  | X |  |
|  | *005/*005 |  | X |  |  |  |  |  |
|  | *005/*001 |  | X | X |  |  |  |  |
|  | *005/*002 |  | X |  | X |  |  |  |
|  | *005/*013 |  | X |  |  | X |  |  |
|  | *005/*007 |  | X |  | X |  | X |  |
|  | *001/*002 |  |  | X | X |  |  |  |
|  | *001/*013 |  |  | X |  | X |  |  |
|  | *001/*007 |  |  | X | X |  | X |  |
|  | *002/*002 |  |  |  | X |  |  |  |
|  | *002/*013 |  |  |  | X | X |  |  |
|  | *002/*007 |  |  |  | X |  | X |  |
|  | *013/*013 |  |  |  |  | X |  |  |
|  | *013/*007 |  |  |  | X | X | X |  |
|  | *007/*007 |  |  |  | X |  | X |  |
