# Supplementary material for: Development of a Novel Multiplex PCR Assay to Detect Functional Subtypes of KIR3DL1 Alleles
Source: PLoS One. 2014 Jun 11;9(6):e99543. doi: 10.1371/journal.pone.0099543 (PMC4053526; doi:10.1371/journal.pone.0099543)
Supplement: Table S2 — Sensitivity and specificity of KIR3DL1 allele subtype assessment. High frequency KIR3DL1 alleles are shown in bold. (DOCX) [file pone.0099543.s002.docx]

**Supplementary Table S2.** Sensitivity and specificity of KIR3DL1 allele subtype assessment. High frequency KIR3DL1 alleles are shown in bold.

| PCR Allele subtype group | KIR3DL1 allele (SBT) | Individuals bearing the allele (n) | KIR3DL1 PCR subtyping performance | |
| --- | --- | --- | --- | --- |
|  |  |  | Sensitivity | Specificity |
| Null | ***004** | 49 | 100% | 100% |
|  | *019 | 2 |  |  |
|  | *072 | 1 |  |  |
| Low-1 | ***005** | 38 | 97.44% | 100% |
|  | *053 | 1 |  |  |
| High-1 | ***001/*016** | 60 | 100% | 100% |
|  | *052 | 1 |  |  |
| High-2 | ***002** | 38 | 100% | 100% |
|  | ***008** | 17 |  |  |
|  | ***009** | 6 |  |  |
|  | ***015** | 17 |  |  |
|  | *018 | 1 |  |  |
|  | ***020** | 5 |  |  |
| KIR3DS1 | ***013** | 55 | 100% | 100% |
|  | *014 | 1 |  |  |
|  | *049N | 3 |  |  |
| Low-2 | ***007** | 10 | 100% | 100% |
|  | *033 | 1 |  |  |
|  |  |  |  |  |
